# Differentiation of imatinib -resistant chronic myeloid leukemia cells with BCR-ABL-T315I mutation induced by Jiyuan Oridonin A

**DOI:** 10.7150/jca.83219

**Published:** 2023-05-05

**Authors:** Yun Xu, Ziting Wang, Lei Zhang, Congying Gao, Fahui Li, Xueming Li, Yu Ke, Hong-Min Liu, Zhenbo Hu, Liuya Wei, Zhe-Sheng Chen

**Affiliations:** 1 School of Pharmacy, Weifang Medical University, Weifang, 261053, China;; 2 School of Pharmacy, Zhengzhou University, Zhengzhou, 450052, China;; 3 Laboratory for Stem Cell and Regenerative Medicine, Affiliated Hospital of Weifang Medical University, Weifang 261042, China;; 4 Department of Pharmaceutical Sciences, College of Pharmacy and Health Sciences, St. John's University, Queens, NY, 11439, USA.

**Keywords:** Chronic myeloid leukemia, BCR-ABL oncogene, T315I mutation, imatinib-resistant BCR-ABL mutations, cell differentiation

## Abstract

Chronic myeloid leukemia (CML) results from BCR-ABL oncogene, which blocks CML cells differentiation and protects these cells from apoptosis. T315I mutated BCR-ABL is the main cause of the resistance mediated by imatinib and second generation BCR-ABL inhibitor. CML with the T315I mutation has been considered to have poor prognosis. Here, we determined the effect of Jiyuan oridonin A (JOA), an *ent*-kaurene diterpenoid compound, on the differentiation blockade in imatinib-sensitive, particularly, imatinib-resistant CML cells with BCR-ABL-T315I mutation by cell proliferation assay, apoptosis analysis, cell differentiation analysis, cell cycle analysis and colony formation assay. We also investigated the possible molecular mechanism by mRNA sequencing, qRT-PCR and Western blotting. We found that JOA at lower concentration significantly inhibited the proliferation of CML cells expressing mutant BCR-ABL (T315I mutation included) and wild-type BCR-ABL, which was due to that JOA induced the cell differentiation and the cell cycle arrest at G0/G1 phase. Interestingly, JOA possessed stronger anti-leukemia activity than its analogues such as OGP46 and Oridonin, which has been investigated extensively. Mechanistically, the cell differentiation mediated by JOA may be originated from the inhibition of BCR-ABL/c-MYC signaling in CML cells expressing wild-type BCR-ABL and BCR-ABL-T315I. JOA displayed the activity of inhibiting the BCR-ABL and promoted differentiation of not only imatinib -sensitive but also imatinib -resistant cells with BCR-ABL mutation, which could become a potent lead compound to overcome the imatinib -resistant induced by inhibitors of BCR-ABL tyrosine kinase in CML therapy.

## Introduction

Chronic myeloid leukemia (CML) is a clonal expansion of the progenitor hematopoietic stem cells arising from the existence of the fusion BCR-ABL oncogene, that represents 15%-20% of the newly diagnosed cases of leukemia patients 1-2. BCR-ABL oncogene encodes a BCR-ABL tyrosine kinase (TK), which triggers different downstream targets including c-Myc, STAT5 and CrkL, which participated in the control of cell differentiation, proliferation, migration and other cellular behaviors 3-5. Hence, BCR-ABL oncogene has been the target for the treatment of patients with CML. For most patients with CML, BCR-ABL tyrosine kinase inhibitors (TKIs) have turned the an inevitably fatal disease into a manageable condition. Imatinib approved by the US FDA (Food and Drug Administration) in 2001 was the mainstay of first-line drug for CML patients as the first-generation TKIs. Therefore, imatinib was a paradigm of targeted therapies, which due to that imatinib inhibits the phosphorylation of BCR-ABL then suppress the activation of BCR-ABL tyrosine kinase 6. However, resistance to imatinib occurs in about 20 - 30% of patients with newly diagnosed CML in chronic phase 7. The mechanisms underlying imatinib resistance are BCR‑ABL kinase-dependent (e.g., point mutations in the BCR-ABL tyrosine kinase domain (TKD) or augmented expression of the BCR-ABL) 8; and BCR-ABL independent (e.g., persistence of quiescent CML stem cell or alterations of efflux influx pumps 9. BCR-ABL kinase mutations has been identified as the dominant cause of acquired imatinib resistance, which are detected in 50-90% of CML patients 10-11. Over 70 different mutant BCR-ABL have been observed in CML patients. The most common point mutations are T315I, E250K, E255K, M351T, Y253F and F359V that account for 60-70% of all mutations in relapsed patients 12. The T315I mutation of BCR-ABL represents approximately 20% of the all mutations. The majority of CML patients with most mutations except T315I can be effectively treated with second-generation TKIs such as dasatinib, nilotinib or bosutinib 13-15. Ponatinib was clinically effective against the T315I mutation 16. However, ponatinib treatment accompanied by adverse side effects limited its clinical utility 17. Fortunately, asciminib was approved by the US FDA to treat CML patients who treated with two prior TKIs or with BCR-ABL-T315I mutation in 2021. The most common adverse reactions of Asciminib include upper respiratory tract infections, decrease of platelet and neutrophil counts, decrease of hemoglobin and increase of triglycerides 18-19. Therefore, the TKI resistance induced by the mutation of BCR-ABL-T315I remains a primary challenge in the management of CML.

CML cells, as well as acute myeloid leukemia cells, are characterized by uncontrolled proliferative lead to impaired terminal differentiation 20. It was found that the human CML K562 cells can undergo differentiation to particular lineages depending on stimuli 21. In addition, the differentiation of BaF3 cells transformed by BCR-ABL can be restored by PP2A activation 22. Moreover, promoting the differentiation of leukemic cells by all-trans retinoic acid, which give a high rate of complete remission, has become a successful treatment for the patients with acute promyelocytic leukemia (M3 subtype of acute myeloid leukemia) 23. Unfortunately, the differentiation-inducing therapy did not been used in CML.

JOA was derived from Isodon rubescens 24. In our previous study, it was found that JOA promoted differentiation of AML cells and has anti-cancer activity against these cells 25-26. It is well known that different tumor cells have different sensitivity to the same drug (compound). Herein, in the current work, we studied whether JOA affect the differentiation blockade of imatinib-resistant CML cells carrying BCR-ABL-T315I mutation. The underlying molecular mechanism responsible for the effect of JOA was also explored. We found that JOA is efficacious in overcoming TKI resistance caused by the mutant BCR-ABL particularly the T315I mutation.

## Materials and methods

### Chemicals and equipment

**Figure 1:**
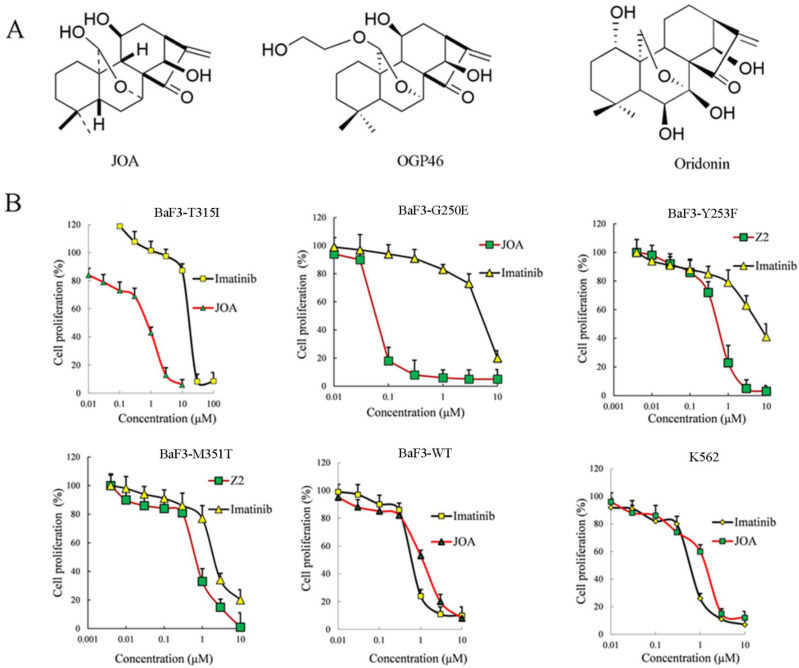
Anti-leukemia efficacy of JOA on BaF3-WT, BaF3-T315I, BaF3-G250E, BaF3-Y253F, BaF3-M351T, BaF3-E255K, BaF3-H296P, BaF3-M244V, BaF3-E255V, BaF3-Q252H, BaF3-F311L, BaF3-F317L, BaF3-F359V and K562cells. (A) structure of JOA, OGP46 and Oridonin. (B) The effect of JOA on the proliferation of cells treated with JOA or imatinib for 72 h. The points with error bars represent the mean ± SD. The figures are representative of three independent experiments done in triplicates.

The purity 98.5% of JOA (molar mass of 348.2) was prepared 24. Figure 1A presents the structure of JOA, its analogues Oridonin 27-28 and OGP46 29. The following chemical, antibodies or reagents are used: fetal bovine serum (FBS), penicillin and streptomycin and RPMI-1640 from Gibco (Carlsbad, USA). Imatinib from sigma-Aldrich (St Louis, MO). FITC anti-human CD13 (catalog MBS670591) from MyBioSource (San Diego, USA), PE anti-human CD14 (catalog 12-0149-42) from eBioscience (San Diego, USA). FITC Annexin Ⅴ kit and propidium iodide (PI)/ RNase reagent from BD biosciences (San Jose, USA). FITC anti-mouse /human CD11b (catalog 101205) antibody from Biolegend (San Diego, USA). MethoCult M3134 (catalog 03134), H4100 (catalog 04100) from Stem Cells Technologies (Vancouver, Canada). SYBR Premix ExTaq reagent from TAKARA Bio Inc. (Otsu, Japan). Trizol® reagent (Invitrogen Life Technologies, CA, USA). The following primary antibodies from Cell Signaling Technology (MA, USA): GAPDH (catalog 5174), c-MYC (catalog 9402), pY177-BCR (phosphorylation of BCR-ABL, p- BCR-ABL, catalog 3901) and BCR-ABL (catalog 3902). FITC anti-mouse CD13 (ab33486), FITC anti-mouse CD14 (ab307635), FITC anti-mouse F4/80 (ab60343), primary antibodies against CCND1 (ab134175) and CDKN2A (ab189034) from Abcam (MA, USA). Flow cytometry analyses were conducted by a flow cytometer of BD Accuri C6 plus (San Jose, USA). RT-qPCR amplification was run on an Applied Biosystems® 7500 Fast from ThermoFisher Scientific Inc. (Waltham, USA).

### Cell lines and cell culture

Human K562 cells (BCR-ABL-native CML), murine BaF3 cells carrying wild-type p210 BCR-ABL (BaF3-WT) and point mutations of p210 BCR-ABL (T315I, E255K, G250E, M351T, Y253F, F359V, E255V, H296P, Q252H, F311L, M244V and F317L) were used. Murine BaF3 cells expressing wild-type or point mutants of BCR-ABL were derived by the transfection of murine hematopoietic cells with a retroviral vector carrying p210 BCR-ABL gene, which were established by Dr. Brian J. Druker (Oregon Health and Science University, USA) 30. Cell lines were cultured with RPMI 1640 containing penicillin/ streptomycin (1%) and FBS (10%) in 5% CO_2_, at 37°C.

### Detection of cell proliferation using MTT assay

The proliferation of BaF3-T315I, BaF3-Y253F, BaF3-G250E, BaF3-M351T, BaF3-E255K, BaF3-H296P, BaF3-M244V, BaF3-E255V, BaF3-Q252H, BaF3-F311L, BaF3-F317L, BaF3-F359V, BaF3-WT and K562 cells was detected by a MTT assay. Cells were seeded for 24 h and cultured with JOA or imatinib for 72 h in 96-well plates. MTT solution (20 μL, 4 mg/mL) was added to each well for incubation 4 h. The plates were centrifuged, then the formazan crystals were obtained and dissolved with DMSO (100 μL). The absorbance value was detected at 570 nm using a microplate reader (Multiskan FC, thermo scientific, USA).

### Apoptosis analysis

BaF3-T315I, BaF3-WT and K562 cells were cultured with of JOA or imatinib for 72 h. Cells were collected, washed with cold PBS for twice, re-suspended in 1× binding buffer and cultured with FITC annexin-V and PI at room temperature in the dark for 30 min. Flow cytometric analysis was used to measure the cell apoptotic percentage.

### Cell morphology analysis

Cells (BaF3-T315I, BaF3-WT and K562) were cultured with JOA and were collected. Cytospin smears were made carefully. The morphology of cells was imaged by light microscopy with Wright-Giemsa stain.

### Cell surface antigen assessment

After JOA treatment, BaF3-T315I, BaF3-WT and K562 cells were collected, centrifuged, and cultured with specific antibody at room temperature for 30 min. The indicated monoclonal antibodies were used to assess the expression of cell surface antigens evaluated by the flow cytometer. The data are presented as mean value of the fluorescence intensity collected from 20,000 cells.

### Cell cycle distribution analysis by flow cytometry

Cells were treated with 2 μM of JOA for a different time period and collected, washed with PBS, fixed with ethanol (70%) at -20 °C then stained with the reagent of PI (50 μg/mL) and RNase A (100 μg/mL). The percentage of cells in different status of cell cycle was determined by the flow cytometer.

### Colony- formation assay

About 5×10^4^ cells were incubated with JOA, the medium containing 2.6% methylcellulose and 10% FBS in 24- well plates (500 μL/well). After 14 days, the colonies composed of more than 50 cells was scored and recorded using an inverted microscope.

### Quantify the genome-wide distribution analysis by mRNA sequencing

To investigate the mRNA expression of genes in cells (BaF3-T315I and K562) incubated with JOA, mRNA sequencing was carried out 25-26. Briefly, quality of extracted RNA was evaluated. 2 μg purified RNA was converted to cDNA library. Sequencing reads were generated and mapped to the genome. mRNAs expression levels were calculated and normalized to transcripts per million. Differentially expressed genes (DEGs) were screened using | log_2_ (fold change) | ≥ 0.58 and p <0.05. Enriched pathways were retrieved from KEGG in BaF3-T315I cells. For K562 cells, it was determined by Gene Set Enrichment Analysis (GSEA) using the C2 category of Molecular Signature Database. The Enriched pathways were deternmined using clusterProfiler package of R software. Signaling pathways were considered as significantly enriched if p value of < 0.05.

### Gene expression analysis by quantitative real-time PCR (qRT-PCR)

Cells (BaF3-T315I and K562) cultured with JOA were harvested to extract total RNA with TRizol reagent. RNA (1 μg) was converted into cDNA using a PrimerScript^TM^ RT reagent kit. qRT-PCR analysis was carried out using SYBR Premix ExTaq reagent. The reactions were performed in triplicate. 2^-ΔΔCt^ method were used to calculate the relative expression quantity of mRNA. The primers were listed as fol1ows: GAPDH (human) forward 5´-TGGGTGTGAACCATGAGAAGT-3´, reverse 5´-TGAGTCCTTCCACGATACCAA-3´ 31; GAPDH (mouse) forward 5´- CAAGGTCATCCATGACAACTTTG-3´, reverse 5´-GTCCACCACCCTGTTGCTGTAG-3´ 32; BCR-ABL (human) forward 5´-TCCGCTGACCATCAATAAGGA-3´, reverse 5´-CACTCAGACCCTGAGGCTCAA-3´ 33; BCR-ABL (mouse) forward 5´-AAGCGCAACAAGCCCACTGTCTAT-3´, reverse 5´-CTTCGTCTGAGATACTGGATTCCT-3´ 34; c-MYC (human) forward 5´-CAGCTGCTTAGACGCTGGATTT-3´, reverse 5´-ACCGAGTCGTAGTCGAGGTCAT-3´ 35 ; c-MYC (mouse) forward 5'-CAGAGG AGGAACGAGCTGAAGCGC-3', reverse 5'-TTATGCACCAGAGTTTCGAAGCTGTTCG-3' 36; CCND1 (human) forward 5´-GAAGATCGTCGCCACCTG-3´, reverse 5'-GACCTCCTCCTCGCACTTCT-3´ 37; CDKN2A (mouse) forward 5'-TTGGCCCAAGAGCGGGGACA-3´, reverse 5'-GCGGGCTGAGGCCGGATTTA-3' 38.

### Western blotting analysis

Total cellular proteins of cells treated with JOA were obtained with lysis buffer. Protein were resolved by sodium dodecyl sulfate polyacrylamide gel electrophoresis and transferred onto polyvinylidene fluoride membranes. The transferred membranes were blocked for 60 min with 5% fat-free dried milk and incubated with specific antibody. Protein bands were visualized by ECL (enhanced chemiluminescence) reagent.

### Statistical analysis

Unless otherwise stated, all experiments were done independently three times. Data with error bars represent mean ± standard deviation (SD). The difference between each treatment group and control group was analyzed based on one-way ANOVA, followed by Dunnett's post-hoc test using SPSS/13.0 software. p < 0.05 was regarded as statistically significant for the difference. (*p < 0.05 and **p < 0.001).

## Results

### JOA significantly reduces the proliferation of CML cells regardless of BCR-ABL mutational forms

As shown in Figure 1B and supplemental Table 1, JOA markedly reduced the proliferation of BaF3 cells harboring 12 mutant BCR-ABL, including T315I, M351T, Y253F, F359V and E255K mutations. Interestingly, JOA was significantly more effective than imatinib (22-fold) in suppressing the proliferation of BaF3-T315I cells. The result reveals that JOA is effective in suppressing the proliferation of imatinib-resistant cells, particularly cells with T315I mutation. The growth of BaF3-WT and K562 cells was greatly inhibited by JOA with a comparable efficacy with imatinib. These results suggest that JOA has an anti-cancer activity against BaF3 cells with mutant BCR-ABL and the CML expressing BCR-ABL. However, compared with imatinib, JOA was less effective against BaF3-F317L and BaF3-F359V cells. Notably, the cell proliferation assay demonstrated that the IC_50_ value of JOA on CML cells for 72 h incubation was significantly lower than that for 48 or 24 h incubation (data not shown in this manuscript). Hence, we choose 72 h incubation time for cell proliferation assay and apoptosis analysis.

### JOA induces minimal apoptosis in CML cells regardless of BCR-ABL mutational forms

**Figure 2:**
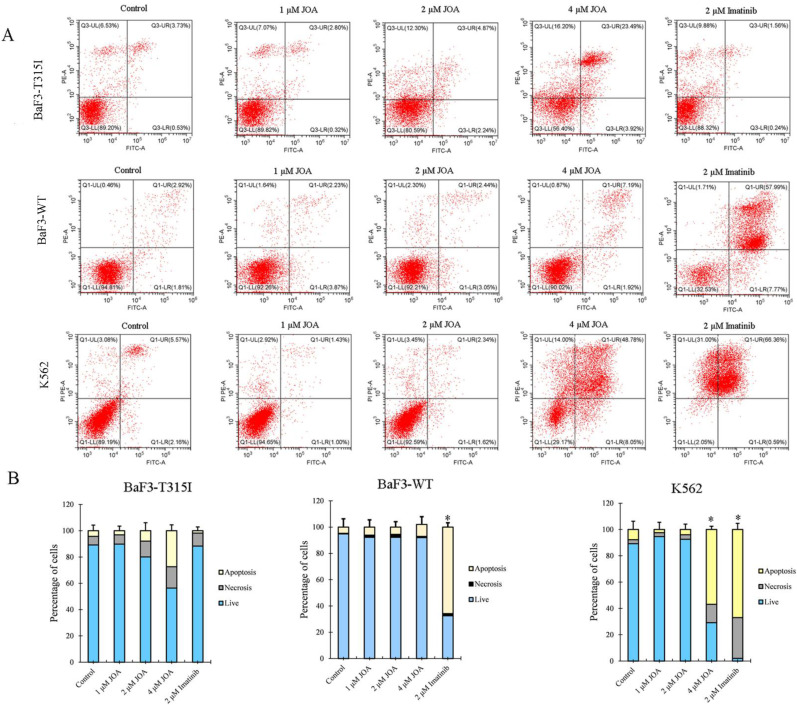
JOA induced minimal signs of apoptosis in BaF3-T315I, BaF3-WT and K562 cells. (A) Analysis of apoptotic cells using the Annexin V-FITC/ PI apoptosis detection kit. (B) Bar graph showing the percentage of apoptotic cells (*P < 0.05 and **P < 0.01). Cells were incubated with 1, 2 or 4 μM of JOA or imatinib (1 or 2 μM) for 72 h.

To explore whether the anti-cancer activity of JOA against BaF3-T315I, BaF3-WT and K562 cells is due to the promotion of cell apoptosis, cells were cultured with JOA or imatinib. As shown in Figure 2A and 2B, 1, 2 or 4 μM of JOA treatment did not lead to prominent apoptosis in BaF3-T315I or BaF3-WT cells. Similarly, a significant change in apoptosis was not found in the K562 cells incubated with JOA (1 or 2 μM). In contrast, imatinib at 2 μM induced significant apoptosis in BaF3-WT and K562 cells. These results demonstrate that the mechanism of how JOA and imatinib inhibit the proliferation of these cells is different. Hence, the underlying mechanism involved in the repression of the proliferative of BaF3-T315I, BaF3-WT and K562 cells cultured with 1 or 2 μM of JOA is not related to cell apoptosis. Hence, 2 μM of JOA is used in the following experiments.

### JOA induces differentiation of CML cells regardless of BCR-ABL mutational forms

**Figure 3:**
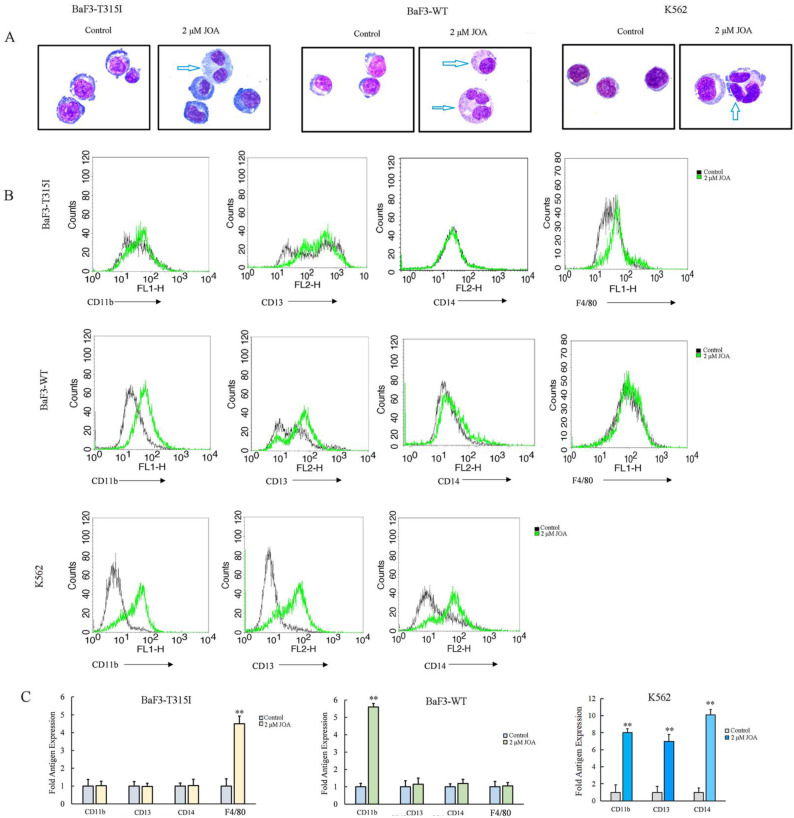
JOA induced cell differentiation in BaF3-T315I, BaF3-WT and K562 cells based on cell morphology and cell membrane markers. (A) Morphological changes of cells stained with Wright-Giemsa captured by oil immersion lens (1000×). (B) The expression of cell surface antigens measured by flow cytometry. (C) Graph bars presenting the mean fluorescence intensity of antigens (**P < 0.01). Cells were incubated with 2 µM of JOA for 72 h.

Since indicated concentration of JOA inhibited CML cell proliferation without promoting apoptosis, we investigated how JOA affect the cell differentiation characterized by morphological features and cell membrane markers in BaF3-T315I, BaF3-WT and K562 cells. As shown in Figure 3A, all three cell lines exhibited visible phenotypic changes and polyploidization. Moreover, JOA treatment caused the elevation of differentiation gene F4/80 in BaF3-T315I cells and an increase of CD11b in BaF3-WT cells. Similarly, JOA significantly up-regulated CD11b, CD13 and CD14 in K562 cells. (Figure 3B). This result indicates that 2 μM of JOA induces the differentiation of BaF3-T315I, BaF3-WT, and K562 cells. These data reveal that the anti-proliferation activity of JOA against CML cells may be due to the promotion of cell differentiation.

### JOA induces G0/G1 phase arrest in CML Cells regardless of BCR-ABL mutational forms

**Figure 4:**
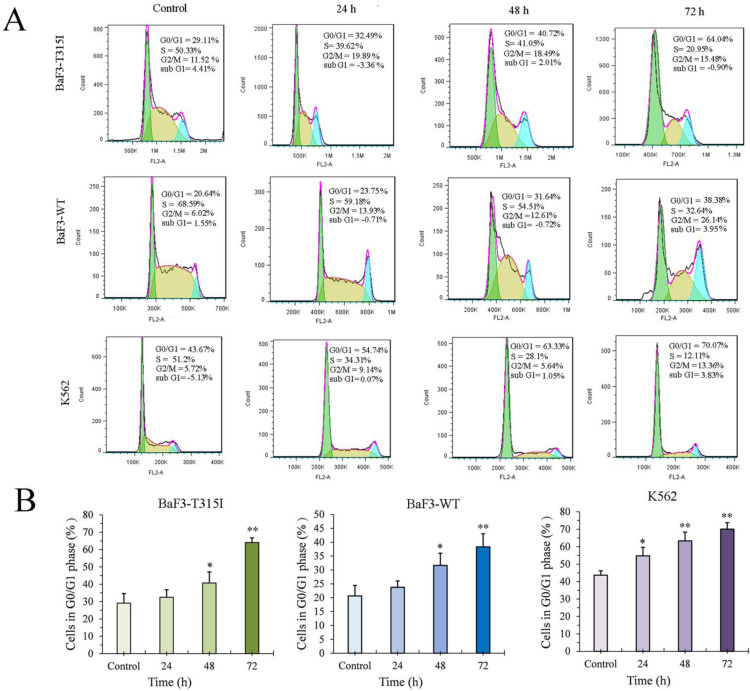
JOA blocked cell cycle exit at G0/G1 phase in BaF3-T315I, BaF3-WT and K562 cells. (A) Cell cycle study by flow cytometry. Representative images of cells based on their profiles in G1, G0/G1, S, and G2/M phases. (B) The percentage of G0/G1 cells presented as a bar graph (*P< 0.05 and **P < 0.01). Cells were incubated with 2 μM of JOA for 24 h, 48 h or 72 h.

As 2 μM of JOA is used to investigate its effect on cell cycle, we performed the cell cycle analysis in different time points by flow cytometry. The percentage of G0/G1 cells was increased in an increasing time in BaF3-T315I, BaF3-WT and K562 cells cultured with JOA of 2 μM (Figure 4). This finding suggests that JOA could induce the G0/G1 phase arrest of CML cells regardless of BCR-ABL mutational forms.

### JOA significantly suppresses colony formation of CML cells regardless of BCR-ABL mutational forms

**Figure 5:**
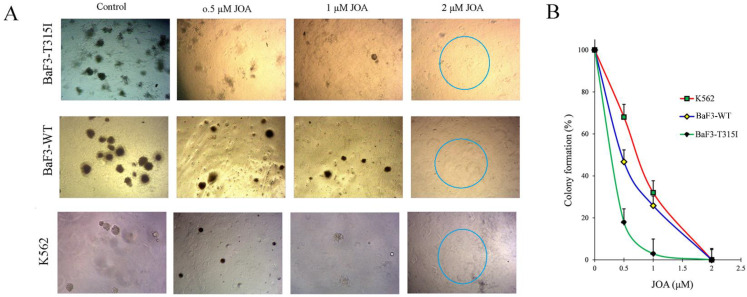
JOA significantly suppressed the colony formation of BaF3-T315I, BaF3-WT and K562 cells. (A) Colony-formation assay in methylcellulose. (B) The effect of different concentrations of JOA on colony formation.

The effect of JOA on the colony formation of CML cells was determined. The total number of colonies was significantly reduced with the increasing concentration of JOA (Figures 5A and 5B). Moreover, colonies could not be found in BaF3-T315I, BaF3-WT and K562 ells treated with 2 µM of JOA, as differentiated cells have had no their ability of colony formation. Notably, BaF3-T315I cells have less colony formation than K562 and BaF3-WT cells cultured with JOA (0.5 or 1 µM). This data was consistent with the fact that the IC_50_ of JOA on BaF3-T315I cells is lower than that on K562 and BaF3-WT cells. These results suggest that JOA could decrease the colony formation of CML cells regardless of BCR-ABL mutational forms, which was due to the promotion of cell differentiation by JOA.

### Cell differentiation promoted by JOA is associated with the inhibition of BCR-ABL and c-MYC

**Figure 6:**
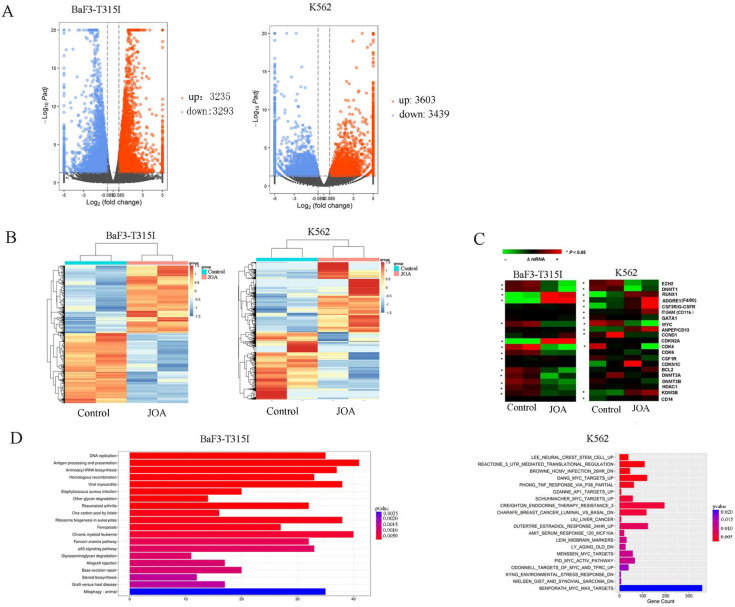
Signaling pathways involved in JOA reduced cell growth in BaF3-T315I and K562 cells. (A) Volcano plots of BaF3-T315I cells and K562 cells. (B) The heatmap of all DEGs. The bars from blue to red denotes the expression levels of DEGs from low to high. (C) The heatmap of differentially expressed genes related with cell differentiation. (*P < 0.05). (D) KEGG or GSEA pathway analyses on all DEGs. BaF3-T315I and K562 cells were incubated with 2 µM of JOA for 48 h.

To examine the underlying molecular mechanism of how JOA induced the differentiation of BaF3-T315I and K562 cells, we analyze the mRNA expression level of genes using mRNA-sequencing. As shown in Figure 6A, it was found that the mRNA expression of 3912 genes were decreased and 3770 genes were increased in BaF3-T315I cells. Similarly, the expression of 3622 genes was up-regulated while that of 3503 genes was down-regulated in K562 cells. These data indicate that the effect of JOA on genes is different (Figure 6B). The heat map of DEGs related with cell differentiation of BaF3-T315I and K562 cells can be seen from Figure 6C. Furthermore, the most optimal pathway related to cell differentiation promoted by JOA was the chronic myeloid leukemia signaling pathway (genes e.g. c-MYC, CDKN2A (cell-cycle inhibitors), CdKN1A, Cdk4, Cdk6, RUNX1, Bcl-2, Sos2, Araf, Kras, E2f2, Smad4 and Chuk were enriched) in BaF3-T315I cells. In addition, the optimal pathways induced by JOA were DANG_MYC_TARGETS_UP SCHUHMACHER_MYC_TARGETS_UP, MENSSEN_MYC_TARGETS, PID_MYC_ACTIV_PATHWAY, BENPORATH_MYC_MAX_TARGETS and ODONNELL_TARGETS_OF_MYC_AND_TFRC_UP signaling pathways in K562 cells (Figure 6D). These pathways are related with the inhibition of c-MYC and its regulating genes Cyclin D1 (CCND1), cell-cycle activators including CDK4 and CDK6 or cell-differentiation related gene ITGAM (CD11b).

**Figure 7:**
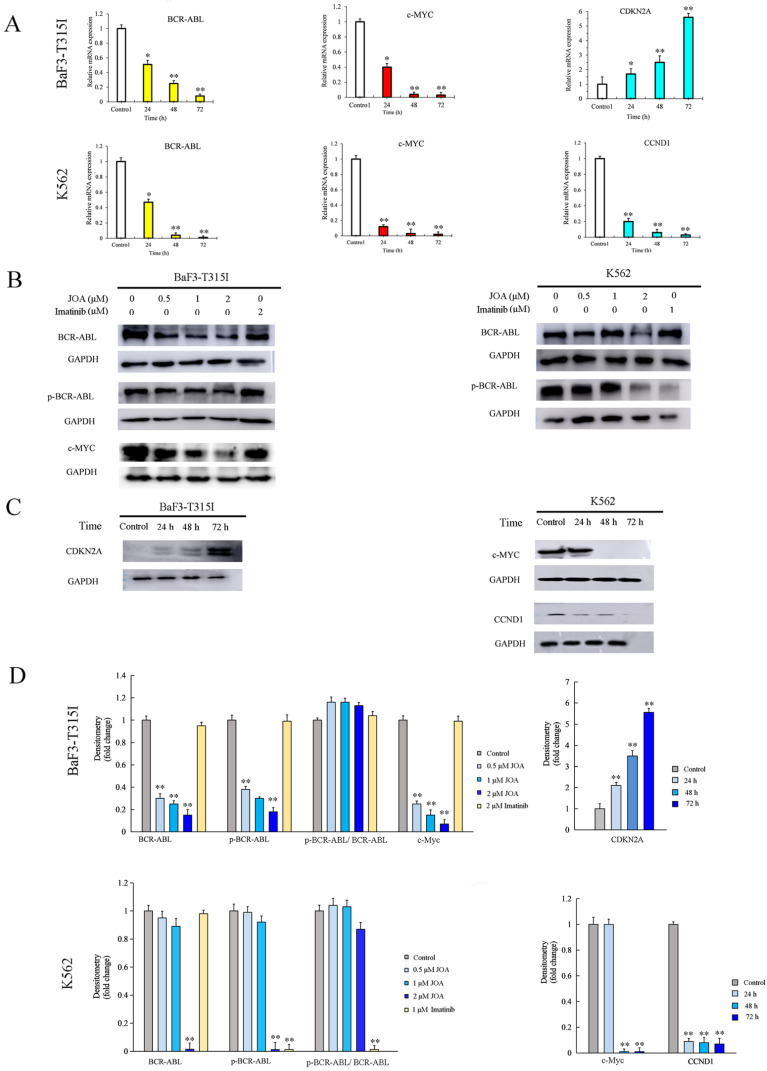
Molecular analysis of the effects of JOA on representative genes in BaF3-T315I cells and K562 cells. (A) The effect of JOA on mRNA expression of BCR-ABL, c-MYC, CCND1 or CDKN2A in K562 and BaF3-T315I cells treated with 2 μM of JOA for 24, 48 or 72 h. (B) Protein expression of BCR-ABL, p-BCR-ABL or c-MYC in both cell lines by western blotting analysis. Cells were incubated with 0.5, 1, or 2 μM of JOA or imatinib (1, or 2 μM) for 72 h. (C) Protein expression of c-MYC, CCND1 or CDKN2A in both cell lines by western blotting analysis. Cells were treated with 2 μM of JOA for 24, 48 or 72 h. (D) Graph bars showing the protein expression quantified by AI600 imager (*P < 0.05 and **P < 0.01).

To validate the expression of DEGs detected by mRNA sequencing analysis, Western blotting and qRT-PCR were performed in BaF3-T315I and K562 cells. It can be seen from Figures 7A, 7B, 7C and 7D, JOA treatment of 2 μM led to the marked decrease of c-MYC and significant increase of CDKN2A at both transcription and protein levels in BaF3-T315I cells. Likewise, the mRNA and protein expression of c-MYC and CCND1 was markedly reduced in K562 cells incubated with JOA. These results fit well with the expression determined by mRNA sequencing in both cell lines.

Since BCR-ABL (P210) mediates the initiation, maintenance and progression of CML and it positively regulate c-Myc expression, we investigated whether JOA reduced the mRNA and protein expression level of BCR-ABL in both BaF3-T315I and K562 cell lines. It was found that the mRNA level of BCR-ABL was also significantly down-regulated in both cell lines incubated with JOA treatment at 2 μM for 72 h (Figure 7A). Similarly, the protein level of BCR-ABL was significantly reduced after treatment of JOA at 2 μM for 72 h in BaF3-T315I and K562 cells cultured with JOA (0.5, 1, or 2 μM) for 72 h (Figures 7B and 7D). These data exhibited that both the transcriptional and protein levels of BCR-ABL were markedly reduced by JOA at 2 μM in both cell lines, whereas imatinib did not alter the expression in BaF3-T315I cells. Hence, the activity of JOA against the proliferation of BaF3-T315I and K562 cells was associated with the decrease of BCR-ABL expression leading to the decrease of the phosphorylation of BCR-ABL (p-BCR-ABL) (Figures 7B and 7D). On the contrary, the anti-cancer effect of imatinib on K562 cells is originated from the decrease of p-BCR-ABL. In addition, imatinib did not change the BCR-ABL or the p-BCR-ABL level in BCR-ABL-T315I cells (Figure 7B and 7D), which was consistent with that imatinib is resistant in the T315I-mutated BCR-ABL cells.

## Discussion

The first- and second-generation TKIs has been widely used clinically for the treatment of CML with tremendous success. However, acquired resistance are observed in most patients treated with these TKIs, leading to poor prognosis. Point mutations of BCR-ABL is a primary cause of imatinib-resistance. Among them, the BCR-ABL-T315I mutation, the most frequently occurring mutation, is resistant to the second-generation TKIs, as well as imatinib. The prognosis of CML patients with this mutation is poor. Small molecule of *Ent*-kaurane diterpenoid such as Oridonin, OGP46, Ponicidin and Phyllostachysin F, have emerged as promising therapeutic agents for various cancers particularly for leukemia 24, 27-29. In our previous study 25-26, it was found that JOA triggered cell differentiation of different AML cell lines via specific signaling pathway. In addition, the great success of all-trans retinoic acid improved the prognosis of acute promyelocytic leukemia. However, the differentiation therapy has not been applied in CML. Hence, in current study, we focus on the effect of JOA on the differentiation blockade in CML cells, especially the CML cells expressing BCR-ABL-T315I. The present work reveals that JOA remarkably reduced the proliferation of CML cells regardless of the mutational forms of BCR-ABL by promoting cell differentiation. Interestingly, it was demonstrated that JOA significantly inhibited the proliferation of 12 BCR-ABL mutant CML cell lines (T315I mutation included). It is well known that differentiation blockade is the hallmark of leukemia. Our findings reveal that JOA could induce the differentiation of CML cells expressing BCR-ABL-T315I, which reveal that JOA may be a potent differentiation inducer for the treatment for CML patients with BCR-ABL mutations.

It was shown that the proliferation of imatinib-sensitive and imatinib-resistant CML cells inhibited by JOA via inducing cell differentiation evidenced by the morphological alteration and the increase of the expression of CD11b, CD13, CD14, CD15 or F4/80 in BaF3-T315I, BaF3-WT and K562 cells. Furthermore, G0/G1 phase block was an accompaniment to cell differentiation of these cells. Importantly, JOA markedly down-regulated the transcriptional and protein levels of BCR-ABL and c-MYC independent of the mutational forms of BCR-ABL. Moreover, the chronic myeloid leukemia signaling pathway was involved in cell differentiation promoted by JOA in BaF3-T315I cells while the differentiation of K562 cells was attributed to c-MYC involved pathway. Interestingly, we found that JOA showed more potency in inhibiting proliferation of K562 cells with the IC_50_ value of 1.44 μM, that were much lower than that of Oridonin which is about 20 μM 27-28. In addition, JOA also exhibited higher anti-proliferation activity against BaF3-T315I cells with the IC_50_ of 0.73 μM, compared with OGP46 (IC_50_ of 1.53 μM). Moreover, JOA has stronger inhibitory activity of the colony formation of BaF3-T315I cells than that of OGP46 (p <0.001). The rate of colony formation was 18% and 2.9%, while it was 77% and 35% when cells were cultured with 0.5 or 1 μM JOA or OGP46, respectively 29. These findings suggest that JOA is efficacious against BCR-ABL-native CML cells and BaF3 cell lines bearing T315I mutations of BCR-ABL via inducing cell differentiation associated with the inhibition of BCR-ABL and c-MYC.

According to the KEGG database, the chronic myeloid leukemia signaling pathway presents that BCR-ABL regulates c-MYC expression. (https://www.kegg.jp/pathway/map05220). Moreover, it has been shown that MYC acts as a cooperative oncogene with the BCR-ABL fusion protein and plays important role in the transformation mediated by BCR-ABL 39-41. Furthermore, the inhibition of BCR-ABL triggering the CML cell differentiation 42-43. In current study, the chronic myeloid leukemia signaling pathway was enriched in BaF3-T315I cells incubated with JOA. In addition, JOA treatment markedly down-regulated the transcription and protein levels of BCR-ABL and c-MYC in BaF3-T315I cells. Therefore, the differentiation of BaF3-T315I cells induced by JOA might be due to the suppressing of BCR-ABL/c-MYC signaling. Similarly, as mentioned above, DANG_MYC_TARGETS_UP, SCHUHMACHER_MYC_TARGETS_UP, MENSSEN_MYC_TARGETS, PID_MYC_ACTIV_PATHWAY, BENPORATH_MYC_MAX_TARGETS and ODONNELL_TARGETS_OF_MYC_AND_TFRC_UP signaling pathways are involved in K562 cells incubated with JOA. Moreover, JOA treatment markedly reduced the transcriptional and protein levels of BCR-ABL and c-MYC in K562 cells. Furthermore, it was revealed that c-MYC is a downstream target gene of BCR-ABL 35, 37-38. Hence, these findings indicated that the anti-cancer activity of JOA against K562 cells was also likely originated from the inhibition of BCR-ABL/c-MYC signaling. Therefore, the mechanism of JOA-mediated differentiation of BaF3-T315I and K562 cells may be originate from the modulation of BCR-ABL/c-MYC signaling, which is different to that caused to the differentiation of AML cells (25-26).

It was well established that the dominant role of c-MYC is to control G1/S transition in cell cycle 44-45. In addition, the transcriptional activity of c-MYC can regulated the mRNA of CCND1 required for cell cycle G1/S transition 46. Similarly, the tumour suppressor CDKN2A are also categorized c-MYC target genes could control the G1/S transition 47-48. Additionally, it was shown that BCR-ABL was involved in control of the G1-S phase transition and degration of the expression of BCR-ABL led to a significant reduce in cell proliferation through down-regulated expression of CCND1 49. Our data were consistent with these findings that JOA treatment significantly down-regulated the expression level of BCR-ABL and c-MYC in BaF3-T315I and K562 cells. Additionally, G0/G1 cell cycle exit was caused by JOA in these cell lines, which is confirmed by the decrease of CCND1 expression in K562 cells and increase of CDKN2A expression in BaF3-T315I cells. This result was consistent with up-regulation of CDKN2A or down-regulation of CCND1 results in G1 phase cell cycle exit 50. Hence, it can be suggested that the cell cycle exit was due to the inhibition of BCR-ABL/c-MYC signaling. Therefore, JOA inhibited induced cell differentiation as well as G0/G1 phase exit in both BaF3-T315I and K562 cells may be originated from the inhibition of BCR-ABL/c-MYC, which was accordance with the finding that cell differentiation is usually coupled with cell-cycle exit 51.

In conclusion, our finding revealed that JOA significantly reduced proliferation and impaired colony formation of not only imatinib-sensitive but also imatinib-resistant CML cells, particularly the BaF3-T315I cells by promoting cell differentiation. The molecular mechanisms of action of JOA is originated from the inhibition of BCR-ABL/c-MYC signaling. Moreover, the anti-leukemia activity of JOA against CML cells is more potent than that of extensively studied compounds such as Oridonin and OGP46. JOA may be potent in surmounting imatinib- resistance caused by the mutations of BCR-ABL (T315I mutation included), indicating that JOA could be worthy of further investigation including in vivo studies. Taken together, our study suggests that JOA may be a lead compound to potentially overcome the limitations of BCR-ABL targeted CML therapy by inhibition of BCR-ABL/c-MYC signaling.

## Supplementary Material

Supplementary table 1.
