# Supplementary material for: Differentiation of imatinib -resistant chronic myeloid leukemia cells with BCR-ABL-T315I mutation induced by Jiyuan Oridonin A
Source: J Cancer. 2023 May 5;14(7):1182–94. doi: 10.7150/jca.83219 (PMC10197941; doi:10.7150/jca.83219)
Supplement: Supplementary file 1 — Supplementary table 1. [file jcav14p1182s1.pdf]

Supplement Table 1. The effect of JOA or imatinib on cell growth and proliferation of K562 and BaF3 cells

BaF3 cell lines	JOA IC50 (μM) ± SD	Imatinib IC50 (μM) ± SD
K562	1.44 ± 0.1	0.68 ± 0.08
BaF3/WT	1.18 ± 0.2	0.75 ± 0.05
BaF3/T315I	0.73± 0.1	16.20 ± 0.7
BaF3/G250E	0.069± 0.06	6.04 ± 0.7
BaF3/Y253F	0.61± 0.04	8.32±1.0
BaF3/M351T	0.75± 0.08	2.27± 0.5
BaF3/E255K	0.059 ± 0.04	0.74 ± 0.07
BaF3/H296P	0.36± 0.03	2.60± 0.4
BaF3/M244V	0.62±0.1	1.81±0.3
BaF3/E255V	0.096±0.03	0.52±0.15
BaF3/Q252H	0.093±0.03	0.29±0.12
BaF3/F311L	0.036±0.04	0.37±0.06
BaF3/F317L	0.43±0.0.6	0.098±0.04
BaF3/F359V	2.35±0.9	0.50±0.13
